# A randomized controlled trial comparing romosozumab and denosumab in elderly women with primary osteoporosis and knee osteoarthritis

**DOI:** 10.1038/s41598-025-05187-7

**Published:** 2025-07-01

**Authors:** Yasumori Sobue, Hironobu Kosugiyama, Shuji Asai, Yoshikazu Ogawa, Minoru Yoneda, Koji Maruyama, Mariko Kaneko, Tomonori Kobayakawa, Kenya Terabe, Mochihito Suzuki, Ryo Sato, Yusuke Ohno, Junya Hasegawa, Takaya Sugiura, Masahiko Ando, Yachiyo Kuwatsuka, Naoki Ishiguro, Shiro Imagama

**Affiliations:** 1 Department of Orthopedic Surgery and Rheumatology, Nagoya University Graduate School of Medicine, 65 Tsurumai-cho, Showa, Nagoya, 466- 8550 Aichi Japan; 2 Department of Orthopedic Surgery, Japanese Red Cross Aichi Medical Center Nagoya Daiichi Hospital, 3-35 Michishita, Nakamura, Nagoya, 453-8511 Aichi Japan; 3 Department of Orthopedic Surgery, Ama Municipal Hospital, 1 Azeta Jimokuji, Ama, 490-1111 Aichi Japan; 4 Department of Orthopedic Surgery, Yoneda Hospital, 1-11-5 Biwajima, Nishi, Nagoya, 451-0053 Aichi Japan; 5 Department of Orthopedic Surgery, Nakatsugawa Municipal General Hospital, 1522-1 Komamba, Nakatsugawa, Gifu, 508-8502 Japan; 6 Department of Orthopedic Surgery, Tokai Hospital, 1-1-1 Chiyodabashi, Chikusa, Nagoya, 464-8512 Aichi Japan; 7 Department of Orthopedic Surgery, National Hospital Organization Higashinagoya National Hospital, 5-101 Umemorizaka, Meito, Nagoya, 465-8620 Aichi Japan; 8 Kobayakawa Orthopedic and Rheumatologic Clinic, 1969 Kunou, Fukuroi, 437-0061 Shizuoka Japan; 9 Department of Advanced Medicine, Nagoya University Hospital, 65 Tsurumai-cho, Showa, Nagoya, 466-8550 Aichi Japan; 10 Aichi Developmental Disability Center, 713-8 Kagiya, Kasugai, 480- 0392 Aichi Japan

**Keywords:** Denosumab, Knee osteoarthritis, Osteophyte, Osteoporosis, Romosozumab, Diseases, Medical research

## Abstract

We compared the efficacy of romosozumab and denosumab in elderly women with primary osteoporosis and knee osteoarthritis in a randomized controlled trial. A total of 112 participants aged 75–90 years were randomized equally into the romosozumab and denosumab groups. Among these, 49 and 52 participants, respectively, who received their initial dose were included in the analysis. The primary outcome was change in lumbar spine (LS)-bone mineral density (BMD) at 12 months in the romosozumab group versus the denosumab group. Secondary outcomes were changes in knee osteophyte development, patient-reported outcomes (PROs), and the incidence of serious adverse events. Mean age of participants was 80.9 years. There was no difference in baseline LS-BMD between the two groups, with a T-score of -2.6. The mean percentage change in LS-BMD at 12 months was significantly higher in the romosozumab group (13.7%) than in the denosumab group (8.5%; *p* = 0.0035). No significant differences were observed in knee osteophyte development and PROs between the two groups. Serious adverse events included a case of mitral regurgitation in the romosozumab group. These findings emphasize the need for refined treatment strategies in high-risk populations, highlighting romosozumab’s benefits and the need to monitor cardiovascular risks.

**Supplementary Information:**

The online version contains supplementary material available at 10.1038/s41598-025-05187-7.

## Introduction

Osteoporosis is a chronic, systemic, and progressive metabolic skeletal disease characterized by low bone mass and microarchitectural deterioration of bone tissue, leading to enhanced bone fragility and a consequent increase in fracture risk^1,2^. The number of patients with osteoporosis is increasing with aging of populations. Approximately 200 million people worldwide are affected by osteoporosis, with 8.9 million fractures occurring each year^3^. Fractures associated with osteoporosis lead to conditions requiring nursing care, increase mortality, and burden healthcare resources^4^. Thus, in the context of rapid global population aging and a projected increase in life expectancy worldwide^5^ the prevention of osteoporosis and associated fractures is very important.

Bone resorption inhibitors such as bisphosphonates and denosumab, as well as bone formation stimulators such as teriparatide, reduce the risk of osteoporosis-related fractures with abundant evidence^6–9^. Denosumab (Prolia, Amgen) is a fully human monoclonal antibody to receptor activator of nuclear factor kappa-B ligand (RANKL). The expression of RANKL in osteoblasts and osteocytes induces osteoclastogenesis, bone resorption, and osteoporosis^10^ and denosumab, administered subcutaneously every six months, inhibits the development and activity of osteoclasts, decreases bone resorption, and increases bone mineral density (BMD) by blocking the binding of RANKL to RANK. Cummings et al.^8^ reported that the risk of new radiographic vertebral fracture was reduced by 68% in the denosumab group, with a cumulative incidence of 2.3% versus 7.2% in the placebo group. On the other hand, romosozumab (Evenity, Amgen and UCB Pharma), a monoclonal antibody that binds and inhibits sclerostin, has a dual effect of increasing bone formation and decreasing bone resorption^11^. Sclerostin, a new therapeutic target for osteoporosis, inhibits Wnt signaling and negatively regulates bone formation in bone homeostasis^12^. Cosman et al.^13^ reported a reduction in the risk of new vertebral fracture by 73% in the group of patients receiving a monthly subcutaneous injection of romosozumab, with a one-year cumulative incidence of 0.5% versus 1.8% in the placebo group. Despite its excellent therapeutic effects, romosozumab may contribute to osteophyte formation by activating the Wnt/β-catenin signaling pathway, which is known to be involved in osteophyte development in osteoarthritis^14–16^. Osteophyte formation is thought to involve endochondral ossification-like processes triggered by hypertrophic chondrocytes in calcified cartilage^17^. This process is regulated by Wnt/β-catenin signaling, which is normally suppressed by sclerostin^16^. When sclerostin is absent or reduced, Wnt/β-catenin signaling is upregulated, promoting chondrocyte hypertrophy and cartilage-to-bone transformation that ultimately underlies osteophyte formation^14^. Given this mechanism, romosozumab-induced suppression of sclerostin may inadvertently promote osteophyte formation through enhanced Wnt/β-catenin signaling activity. Thus, the safety of romosozumab, including its potential to induce osteophyte formation in humans, needs to be verified.

No randomized controlled trials have directly compared the effects of romosozumab and denosumab, with a focus on elderly women with primary osteoporosis and knee osteoarthritis—a population at risk not only for fragility fractures but also for joint degeneration. While osteoarthritis may not necessarily alter pharmacologic treatment decisions for osteoporosis, its co-existence introduces additional clinical considerations. Specifically, osteoarthritis is associated with structural joint changes and pain that may interact with bone quality, physical function, and quality of life (QOL). Thus, it becomes important to consider whether osteoporosis treatments might influence not only bone density but also joint-related outcomes. Given that romosozumab may affect joint structures through Wnt/β-catenin signaling, the present study aimed to clarify the efficacy and safety of these therapies in a clinically relevant population.

## Results

### Participant characteristics

A total of 112 participants were enrolled, with 101 included in the final analysis. At 12 months, 38 participants in the romosozumab group and 46 in the denosumab group completed follow-up. Major reasons for dropout included adverse events such as injection site reactions and cardiopulmonary events.

**Fig. 1:**
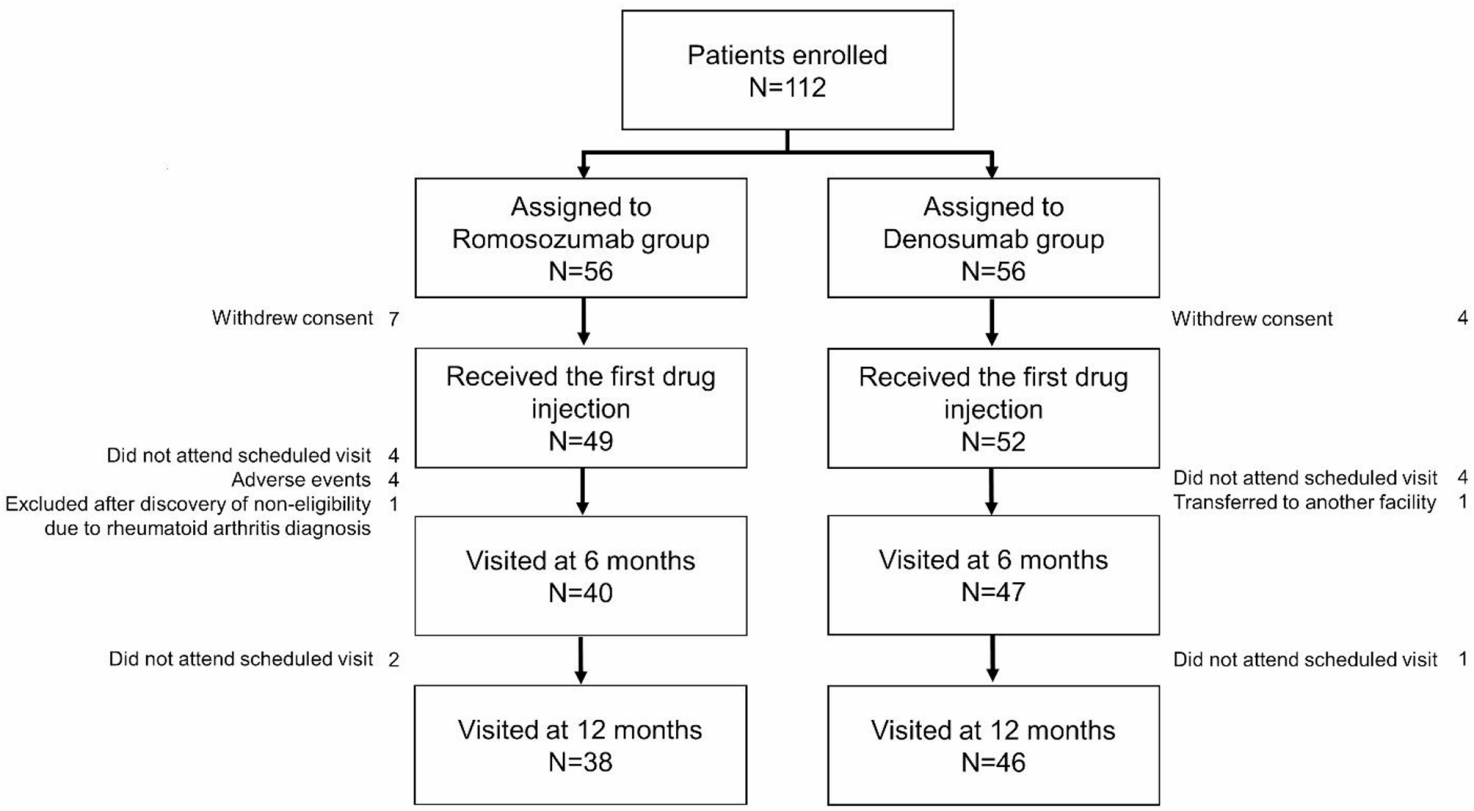
Flowchart of participant selection depicting the number of patients enrolled at each stage of the study.

The initial 112 participants, enrolled between January 2020 and January 2023, were evenly divided into the romosozumab and denosumab groups (*n* = 56 each) (Fig. 1). Among these, 49 participants from the romosozumab group and 52 from the denosumab group who received their first dose were included in the safety and efficacy analyses. At the 12-month time point, 38 and 46 participants were remaining in the romosozumab and denosumab groups, respectively. Participants who dropped out included four participants in the romosozumab group who developed adverse events (one event each of mitral regurgitation and respiratory failure and two events of injection site reactions). Table 1 presents data from 101 participants treated with romosozumab or denosumab. Mean age was 80.9 years (romosozumab group: 81.3 years vs. denosumab group: 80.4 years; *p* = 0.240), and mean body mass index (BMI) was 21.1 kg/m² (20.7 kg/m^2^ vs. 21.5 kg/m^2^; *p* = 0.200). No significant difference was observed in baseline LS-bone mineral density (BMD) between the two groups, with a mean T-score of -2.6 (*p* = 0.870). Thus, the two groups were well-matched in terms of baseline characteristics for comparisons.

**Table 1:** Demographic and clinical characteristics of patients.

Variable	Total (*n* = 101)	Romosozumab (*n* = 49)	Denosumab (*n* = 52)	*p* value
Age (years)	80.9 (3.9)	81.3 (3.8)	80.4 (4.1)	0.240
BMI (kg/m^2^)	21.1 (2.9)	20.7 (3.0)	21.5 (2.8)	0.200
T-score				
Lumbar spine	-2.6 (1.3)	-2.6 (1.3)	-2.6 (1.3)	0.870
Total hip	-2.9 (1.0)	-3.0 (1.2)	-2.8 (0.9)	0.270
Femoral neck	-3.4 (0.8)	-3.5 (0.8)	-3.2 (0.8)	0.084
Prior use of active vitamin D				
Yes	14 (14)	6 (12)	8 (15)	0.550
No	86 (85)	43 (88)	43 (83)	
Missing	1 (1)	0 (0)	1 (2)	
Smoking				
Currently smoking	4 (4)	2 (4)	2 (4)	0.910
Not smoking	89 (88)	43 (88)	46 (88)	
Previously smoking	5 (5)	2 (4)	3 (6)	
Missing	3 (3)	2 (4)	1 (2)	
Brinkman index	350.6 (285.5)	475.0 (370.8)	251.0 (178.9)	0.270
Drinking				
Yes	3 (3)	3 (6)	0 (0)	0.160
No	93 (92)	43 (88)	50 (96)	
Missing	5 (5)	3 (6)	2 (4)	
Exercise habit				
Yes	36 (36)	21 (43)	15 (29)	0.140
No	61 (60)	25 (51)	36 (69)	
Missing	4 (4)	3 (6)	1 (2)	
Daily exercise duration (min)	37.7 (26.1)	36.1 (26.7)	40.0 (26.1)	0.670
Whether patients have ever had hip fracture				
Yes	6 (6)	2 (4)	4 (8)	0.430
No	91 (90)	44 (90)	47 (90)	
Missing	4 (4)	3 (6)	1 (2)	
Blood examination				
Serum-corrected calcium (mg/dL)	8.3 (2.0)	8.1 (2.1)	8.4 (2.0)	0.590
Serum phosphorus (mg/dL)	3.6 (0.5)	3.6 (0.4)	3.6 (0.5)	0.940
Serum creatinine (mg/dL)	0.7 (0.1)	0.7 (0.1)	0.7 (0.1)	0.410
Intact PTH (µg/mL)	48.1 (20.9)	47.2 (16.9)	49.0 (24.0)	0.670
PINP (µg/L)	73.9 (40.1)	80.0 (50.6)	68.5 (27.4)	0.180
TRACP-5b (mU/dL)	596.3 (216.8)	609.8 (233.9)	584.4 (202.1)	0.570
25OHD (ng/mL)	20.4 (16.8)	21.0 (19.1)	19.8 (14.8)	0.720

Continuous variables, mean (standard deviation); categorial variables, n (%); BMI, body mass index; intact PTH, intact parathyroid hormone; P1NP, procollagen type 1 N-terminal propeptide; TRACP-5b, tartrate-resistant acid phosphatase isoform 5b; 25OHD, 25-hydroxyvitamin D.

### Summary of key primary and secondary outcome findings

**Fig. 2:**
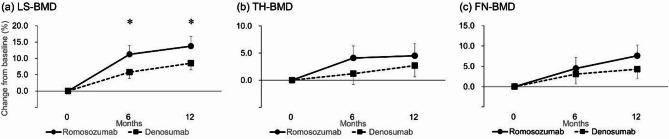
Mean percentage change from baseline in (a) lumbar spine (LS)-bone mineral density (BMD), (b) total hip (TH)-BMD, and (c) femoral neck (FN)-BMD. Error bars represent 95% confidence intervals. *P < 0.01; comparison between the romosozumab and denosumab groups.

**Fig. 3:**
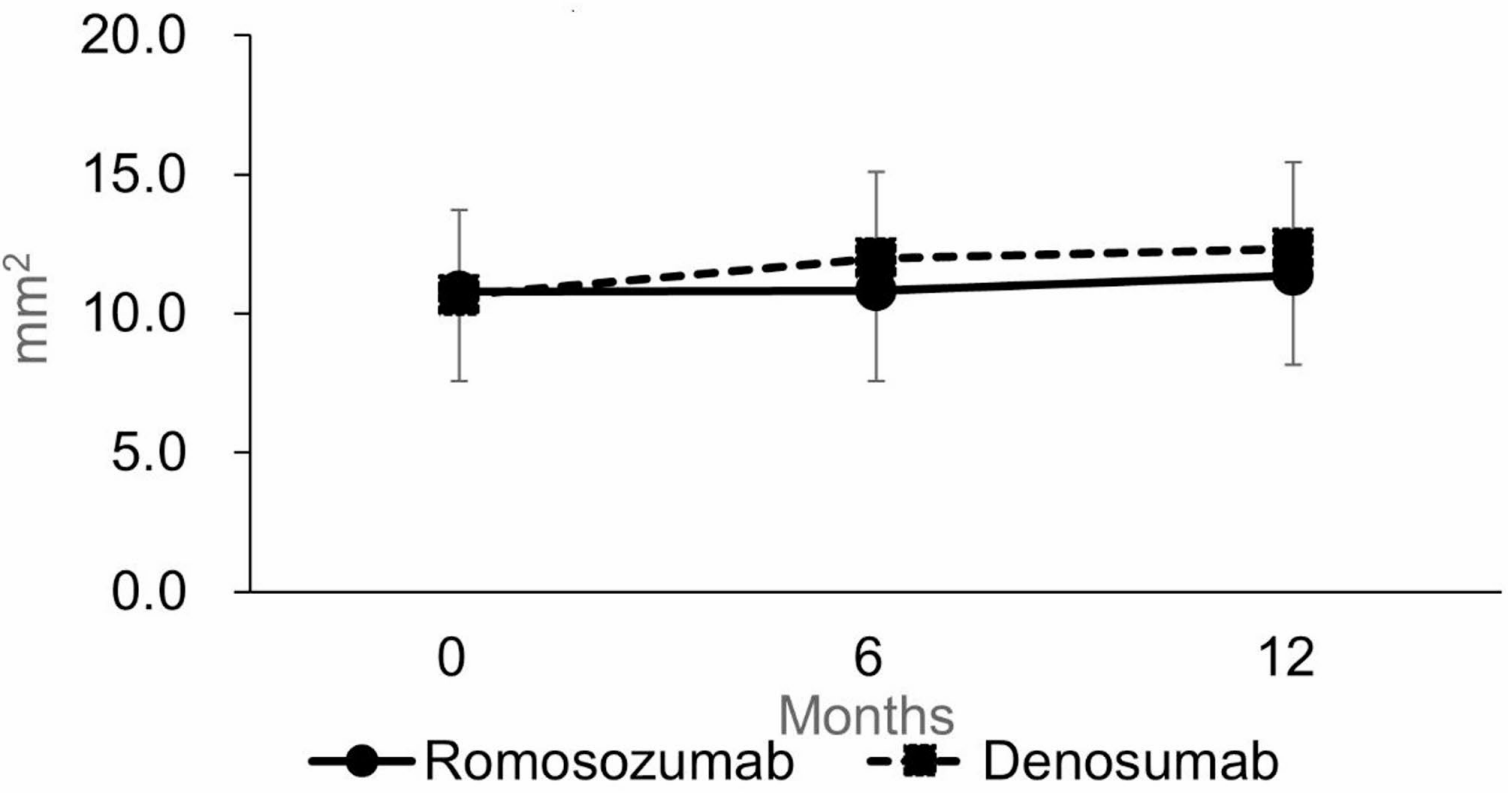
Estimated mean knee osteophyte area. Error bars represent 95% confidence intervals. No significant difference was observed between the romosozumab and denosumab groups.

**Fig. 4:**
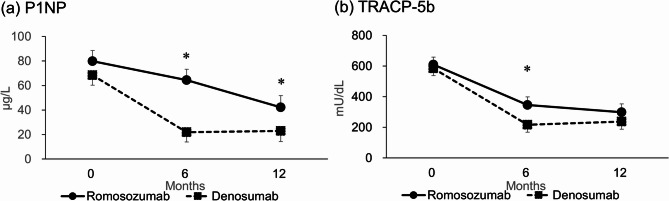
Estimated mean change in (a) procollagen type 1 N-terminal propeptide (P1NP) and (b) tartrate-resistant acid phosphatase isoform 5b (TRACP-5b). Error bars represent 95% confidence intervals. *P < 0.001; comparison between the romosozumab and denosumab groups.

- Lumbar Spine (LS-)BMD at 12 months (Primary Outcome) (Fig. 2a):
  - Romosozumab group: +13.7% (95% confidence interval (CI), 10.8–16.7) vs. Denosumab group: +8.5% (6.5–10.5), significant difference (*p* = 0.0035).

- LS-BMD at six months (Fig. 2(a)):
  - Romosozumab group: +11.2% (8.4–14.0) vs. Denosumab group: +5.8% (3.9–7.7), significant difference (*p* = 0.0014).

- Total Hip (TH-)BMD and Femoral Neck (FN-)BMD (Fig. 2(b), 2(c)):
  - At six months:
    - TH-BMD: Romosozumab group + 4.1% (1.8–6.3) vs. Denosumab group + 1.2% (− 0.9–3.2), *p* = 0.2406.
    - FN-BMD: Romosozumab group + 4.5% (1.9–7.2) vs. Denosumab group + 3.1% (0.7–5.4), *p* = 0.8435.
  - At 12 months:
    - TH-BMD: Romosozumab group + 4.5% (2.2–6.8) vs. Denosumab group + 2.7% (0.6–4.8), *p* = 0.6457.
    - FN-BMD: Romosozumab group + 7.6% (4.9–10.2) vs. Denosumab group + 4.3% (2.0–6.7), *p* = 0.2790.

- Knee Osteophyte Area (Fig. 3):
  - No significant differences at six or 12 months (*p* = 0.6430, 0.3852).
  - Baseline calcium (Ca) level was a significant predictor at 12 months (*p* = 0.039; odds ratio 0.766, 95% CI 0.595–0.986).

- Patient-reported Outcomes (PROs) and Grip Strength (Supplementary Fig. 1):
  - No significant differences were observed at six or 12 months in any PROs, including pain visual analog scale (VAS), patient’s global assessment VAS, physician’s global assessment VAS, Knee Japanese Orthopedic Association (JOA) score, Oxford Knee Score (OKS), Functional Assessment of Chronic Illness Therapy (FACIT)-fatigue scale, EQ-5D-5 L (EQ-5D), the second edition of the Beck Depression Inventory (BDI-2), SF-36, or grip strength.
  - Pain VAS decreased slightly over 12 months:
    - Romosozumab group: − 3.4 mm (27.4 → 24.0).
    - Denosumab group: − 2.1 mm (25.6 → 23.5).

- Serum markers and bone turnover markers (BTMs):
  - Ca, phosphorus (P), creatinine (Cre), and intact parathyroid hormone (PTH): No significant differences at six or 12 months.
  - N-terminal propeptide of type 1 collagen (P1NP) (Fig. 4(a)): Significant differences at six and 12 months (*p* < 0.0001, *p* = 0.0003).
  - Tartrate-resistant acid phosphatase (TRACP)-5b (Fig. 4(b)): Significant at six months (*p* < 0.0001) but not at 12 months (*p* = 0.1150).

- Radiographic changes and fractures:
  - New vertebral fractures:
    - Romosozumab group: 0%.
    - Denosumab group: 5.0% (six months), 8.8% (12 months).
    - No significant differences.
  - New osteophytes: 0% in both groups.

- Arthritis exacerbation or total knee arthroplasty:
  - None in either group.

- Serious adverse events (Table 2):
  - Romosozumab group: Mitral regurgitation, respiratory failure (each 2.0%).
  - Denosumab group: Pulmonary non-tuberculous mycobacterial infection (1.9%).
  - No significant differences between groups were found at up to six months (*p* = 0.141), from six to 12 months (*p* = 0.329), or over the entire period (*p* = 0.523).

**Table 2:** Serious adverse events during treatment.

	Romosozumab (*n* = 49)	Denosumab (*n* = 52)
Serious adverse events		
Mitral regurgitation	1 (2.0)	0 (0.0)
Respiratory failure	1 (2.0)	0 (0.0)
Pulmonary non-tuberculous mycobacterial infection	0 (0.0)	1 (1.9)

Categorial variables, n (%).

### Description of primary and secondary outcome findings

An explanation of the primary and secondary outcomes is provided below to complement the structured summary above. The primary outcome of the study was the percentage change in LS-BMD at 12 months, which was significantly greater in the romosozumab group (13.7% [10.8–16.7]) than in the denosumab group (8.5% [6.5–10.5]; *p* = 0.0035) (Fig. 2(a)). The mean percentage change in LS-BMD at six months was also significantly greater in the romosozumab group (Fig. 2(a)). In contrast, no significant differences were observed between the groups for changes in TH-BMD or FN-BMD at either six or 12 months (Figs. 2(b) and 2(c)). Similarly, no significant differences in knee osteophyte area were found between the groups at either time point (Fig. 3).

A multivariate analysis identified lower baseline Ca levels as a significant risk factor for increased osteophyte area at 12 months. PROs and grip strength showed no significant differences at six or 12 months (Supplementary Fig. 1). Among serum markers and BTMs, P1NP levels were significantly different at both six and 12 months (Fig. 4(a)), while TRACP-5b levels differed only at six months (Fig. 4(b)). The incidence of new vertebral fractures was lower in the romosozumab group but did not reach statistical significance. No cases of arthritis exacerbation or total knee arthroplasty occurred. Serious adverse events included mitral regurgitation and respiratory failure in the romosozumab group, and pulmonary non-tuberculous mycobacterial infection in the denosumab group, with no significant differences observed between groups over the entire study period (Table 2).

## Discussion

This randomized controlled trial was the first to directly compare the efficacy of romosozumab and denosumab in terms of change in LS-BMD over a 12-month period in elderly women with primary osteoporosis and knee osteoarthritis. We found a 13.7% increase in LS-BMD with romosozumab, which was significantly higher than the 8.5% increase observed with denosumab. These results, showing an increase in LS-BMD with the use of romosozumab, are consistent with outcomes from major clinical trials. The FRAME trial reported a significantly improved LS-BMD after 12 months of romosozumab treatment, with a 13.3% increase compared with placebo^18^. Similarly, the ARCH trial reported a 13.7% increase after one year of romosozumab treatment, which was significantly higher compared with the alendronate group^19^. The comparative effectiveness highlighted in the present study provides valuable clinical insight into the differential impact of these treatments on bone density, especially in a head-to-head RCT setting.

Regarding the safety profile, it is important to note that serious adverse events were more frequent, albeit not significantly, in the romosozumab group (2/49 participants) than in the denosumab group (1/52 participants). One of the two serious adverse events observed in the romosozumab group was mitral regurgitation, which is classified as a cardiovascular event. Romosozumab is contraindicated in patients with a history of significant cardiovascular events within the past year, primarily due to findings from the ARCH trial, which showed a higher incidence of ischemic heart and cerebrovascular events in the treatment group^19^. However, a real-world study showed that patients receiving romosozumab treatment experienced fewer major adverse cardiovascular events compared with those receiving PTH analog therapy during a one-year follow-up^20^. This suggests a potentially safer cardiovascular profile than initially feared. Nevertheless, it is crucial to proceed with particular caution when treating high-risk patients, such as those with advanced age or a pre-existing history of cardiovascular disease. Clinicians should carefully weigh the benefits and risks of romosozumab, ensuring thorough cardiovascular evaluation and monitoring throughout the treatment period to mitigate potential adverse effects.

We also compared the area of knee osteophytes between the romosozumab and denosumab groups. The results indicated no significant differences between the two groups in terms of osteophyte development in the knee region and the development of osteoarthritis. Denosumab, which has bone resorption inhibiting properties, might offer benefits in osteoarthritis treatment by targeting subchondral bone remodeling^21^. The subchondral bone plays a crucial role in joint integrity, and its stability affects the health of the overlying cartilage. By inhibiting osteoclast activity, denosumab can stabilize the subchondral bone, thereby potentially reducing stress on the cartilage and slowing osteoarthritis progression^21^. In the present study, however, no significant differences were observed in the development of osteoarthritis when compared directly with romosozumab, indicating that both treatments have similar effects on subchondral bone remodeling. Romosozumab binds to and inhibits sclerostin, which is widely viewed as an osteocyte-specific protein but is also expressed in articular chondrocytes. Sclerostin expression is focally increased in cartilage in osteoarthritis but is decreased in the subjacent subchondral bone^22^. The substudy of the FRAME trial that assessed the effects of romosozumab on cartilage function raised concerns about whether its inhibition of sclerostin could negatively impact cartilage integrity and accelerate osteoarthritic processes due to potential microarchitectural changes in the joints caused by increased bone density^23^. While concerns exist about romosozumab possibly exacerbating osteoarthritis, its direct effects on osteoarthritis are limited^23^ suggesting a complex interplay between bone density enhancement and joint health and a particular need for caution when prescribing treatment for osteoporosis in patients with concurrent joint disorders.

A comparison of the effects of romosozumab and denosumab on PROs in patients with osteoporosis revealed no significant differences. Both treatments are primarily designed to increase bone density and reduce the risk of fractures in patients with osteoporosis. Meanwhile, their direct effects on joint pain and overall QOL, which are crucial aspects of osteoarthritis management, are unclear. Previous studies on osteoporosis treatments reported mixed results regarding joint pain and functional limitations^24,25^. In particular, one study suggested that women with lower bone mineral density tend to experience greater osteoarthritic pain and disability, possibly due to reduced physical activity and compromised bone quality^24^. However, in the present study, only modest numerical improvements in pain VAS scores were observed. This underscores the need for further investigation into how osteoporosis treatments might also support overall musculoskeletal health beyond just bone density improvements. Thus, although the present study found no significant differences in PROs between the romosozumab and denosumab groups, a further study is warranted to explore in more detail the broader implications of their use in the management of pain and physical functioning in patients with osteoporosis.

There are limitations in this study that are worth noting for accurate interpretation of the findings. First, although sufficient statistical power was achieved, the small sample size of the study limited the breadth of data available for analysis. In addition, the high dropout rate might have introduced bias, as participants who remained in the study might not have fully represented the initial population. Furthermore, the present study did not evaluate the extent of improvement in PROs in each group. Another limitation is the composition of the trial, which predominantly included elderly individuals. This raises concerns about the reliability and consistency of PROs due to potential cognitive impairment in this age group. Moreover, the one-year study period may not have been sufficient to conclusively assess the long-term effects of romosozumab and denosumab on osteoarthritis progression. A future study with a longer follow-up period will be necessary to fully understand the impact and durability of these treatments.

In conclusion, the present randomized controlled trial demonstrated that romosozumab can achieve a significantly greater increase in LS-BMD compared with denosumab at both six months and 12 months in elderly women with primary osteoporosis and knee osteoarthritis, highlighting the efficacy of romosozumab in improving BMD in this patient population. It was also confirmed that romosozumab and denosumab exert similar effects on osteoarthritis progression and PROs, with no significant differences in knee osteophyte development and PROs between the two groups. These findings will provide valuable insights into tailored therapeutic strategies in high-risk populations, such as elderly patients and those with a pre-existing history of cardiovascular disease, and suggest that clinicians should carefully weigh the benefits and risks of romosozumab while ensuring thorough cardiovascular evaluation and monitoring throughout the treatment period to mitigate potential adverse effects.

## Methods

### Study design

The Total REview of Anti-Sclerostin antibody Use Related to Elderly people (TREASURE) study was a randomized, multicenter, open (masking not used), active-controlled, and parallel-assignment trial for treatment purposes (jRCT1041190081)^26^. Women who met the selection criteria of the TREASURE study were randomly assigned in a 1:1 ratio to receive subcutaneous injections of either romosozumab 210 mg once a month or denosumab 60 mg once every six months for 12 months. The case registration assignment web system constructed by the Department of Advanced Medicine, Nagoya University Hospital, was used. In principle, all patients were administered eldecalcitol (0.75 micrograms orally once daily, dose reduced to 0.5 micrograms once daily depending on symptoms). The randomization was stratified by facility, age (< 82.5 vs. ≥82.5), BMI (< 23 vs. ≥23), T-score (<-2.5 vs. ≥-2.5), and use of eldecalcitol before the start of the study (yes vs. no) using a minimization method.

This study was initially registered on December 16, 2019, with an expected enrollment of 100 patients. However, the higher than anticipated dropout rate identified through monitoring was suspected to compromise the statistical power of the study. Thus, the sample size was increased by 10 patients to a total of 110 patients on July 1, 2022, and further, to a total of 120 patients on January 15, 2023. Additionally, on February 14, 2024, the study period was extended from the original end date of March 31, 2024, to March 31, 2025. Changes were also made to the principal investigator and responsible physician due to relocation or retirement.

### Study oversight and ethical statement

The study protocol was registered in the Japan Registry of Clinical Trials (jRCT1041190081) on 19/12/2019 and approved by Nagoya University Clinical Research Review Board (CRB4180004) on 4/10/2019. With the approval of the CRB, research permits were obtained for all participating facilities. All patients provided written informed consent. Research expenses were covered by Nagoya University Hospital Funding for Clinical Research. Patient anonymity was maintained during data collection, and the security of personal information was strictly controlled. The study was conducted in accordance with the World Medical Association of Helsinki ethical principles for medical research involving human subjects.

### Patients

Elderly women aged 75–90 years with primary osteoporosis and knee osteoarthritis (Kellgren–Lawrence Grade II or III^27^ on either side) were eligible for participation. Primary osteoporosis was defined as follows: (1) By differential diagnosis, excluding secondary osteoporosis and other diseases that result in low bone mass; (2) Without osteoporotic fractures (lumbar or hip BMD ≤ 70% of young adult mean (YAM), or T-score ≤-2.5); and (3) With osteoporotic fracture (hip or vertebral fracture, or other osteoporotic fracture, and lumbar or hip BMD ≤ 80% of YAM referred to the 2015 Japanese Guidelines for Prevention and Treatment of Osteoporosis^28^). Patients had to have at least two lumbar vertebrae (L1-L4) and at least one hip that could be evaluated by means of dual-energy X-ray absorptiometry (DXA). Patients who had previous bisphosphonate, teriparatide, romosozumab, or denosumab use, strontium use (for other osteoporosis drugs or treatments that affect bone metabolism, a certain wash-out period was allowed), current or previous steroid use (more than three months and 5 mg), previous solid organ or bone marrow transplants, previous osteonecrosis of the jaw, glomerular filtration rate (GFR) ≤ 45 mL/min/1.73 m^2^ hypercalcemia or hypocalcemia defined as albumin-adjusted serum calcium outside the normal range, and cardiovascular events within 1 year were excluded.

### Procedures

The following demographic data were recorded at baseline (before treatment): age, height, weight, BMI, past medical history including fragility fractures, family history, comorbidity, lifestyle (smoking, alcohol intake, exercise habits), concomitant drugs, and prior treatment with eldecalcitol. PROs (see the next section for details) and grip strength were recorded at baseline, six months, and 12 months, and at the time of exacerbation of arthritis, unilateral total knee arthroplasty, and discontinuation of the study. Exacerbation of arthritis was defined as an increase in pain VAS by ≥ 20 mm and requiring an intraarticular injection of hyaluronic acid or local anesthesia. The level of serum 25-hydroxyvitamin D (25(OH)D) was measured at baseline. Levels of serum Ca, P, Cre, and intact PTH were measured at baseline, six months, and 12 months, and if possible, at discontinuation of the study. For BTMs, levels of serum P1NP and serum TRACP-5b were recorded at baseline, six months, and 12 months, and if possible, at discontinuation of the study. LS-, TH-, and FN-BMD values were recorded to evaluate the treatment effect of osteoporosis at baseline, six months, and 12 months, and if possible, at discontinuation of the study. BMD was measured by DXA (Hologic or Lunar). Radiographs of the lumbar spine and front standing knee were obtained at baseline, six months, 12 months, and if possible, at discontinuation of the study. BMD and radiographs were available within four weeks before the start of treatment. Radiographs of vertebral fractures were assessed using the Genant grading scale (grades 0 to 3)^29^.

Adverse events were evaluated in accordance with the Common Terminology Criteria for Adverse Events v5.0 Japanese version (CTCAE v5.0-JCOG). In this study, a serious adverse event was defined as any adverse event of severity Grade 3 or higher.

The criteria for discontinuation of the study were as follows: difficulty in continuing the study due to exacerbation of complications or adverse events; a delay in administration of romosozumab or denosumab by ≥ 1 week from the scheduled administration date; and total knee arthroplasty (however, in the case of one-sided total knee arthroplasty, evaluation of the other side was to be continued).

The following treatments were prohibited during the study period: use of osteoporosis drugs other than romosozumab, denosumab, and eldecalcitol; use of strontium, tibolone, and cinacalcet; androgen removal or hormone removal therapy; use of drugs that can reduce bone mass, such as steroids; and intra-articular injection of steroids.

### Primary and secondary outcomes

The primary outcome was change in LS-BMD at 12 months in the romosozumab group versus the denosumab group. Secondary outcomes were changes in TH-BMD, FN-BMD, knee osteophyte area, PROs, grip strength, and BTMs. In addition, differences in the proportions of patients with new vertebral fractures, exacerbation of arthritis, and adverse events were compared between the romosozumab and denosumab groups.

PROs included the patient’s assessment of pain VAS, patient’s global assessment of VAS (answer to the question “How are you feeling today?”), physician’s global assessment of VAS (from 0 to 100 mm), patient’s JOA score^30^ OKS^31,32^ FACIT-fatigue scale^33^ SF-36^34,35^, EQ-5D^34,35^ and BDI-2^38^. The Knee JOA score was used to assess the severity of knee osteoarthritis in the range of 0 to 100 points, with 100 points indicating the best condition, and consisted of four domains (pain and walking ability, pain and stair-climbing ability, flexion angle and intense and/or severe contraction, and swelling)^30^. The OKS (Japanese version) was also used to evaluate knee osteoarthritis severity and ranged from 0 to 48 points, with 48 points indicating the best outcome^31,32^. The FACIT-fatigue scale (version 4, Japanese) consists of 13 questions on fatigue, with scores ranging from 0 to 52 points; lower scores indicate higher fatigue and lower QOL^33^. The SF-36 (Japanese version 2.0) was used to evaluate QOL^36,37^; the eight scales and two summary measures of the SF-36, the Physical Component Summary (PCS) and Mental Component Summary (MCS), are expressed in the range of 0 to 100 points. The EQ-5D (Japanese version), which was also used to evaluate QOL, consists of five dimensions (mobility, self-care, usual activities, pain/discomfort, and anxiety/depression) reported at five levels of severity from 0 (death) to 1 (full health)^36,37^. The BDI-2 (Japanese version) consisting of 21 items was used to evaluate the level of depressive symptoms, with scores ranging from 0 to 63 points (scores ≥ 14 indicate the presence of at least mild to moderate symptoms of depression)^38^.

**Fig. 5:**
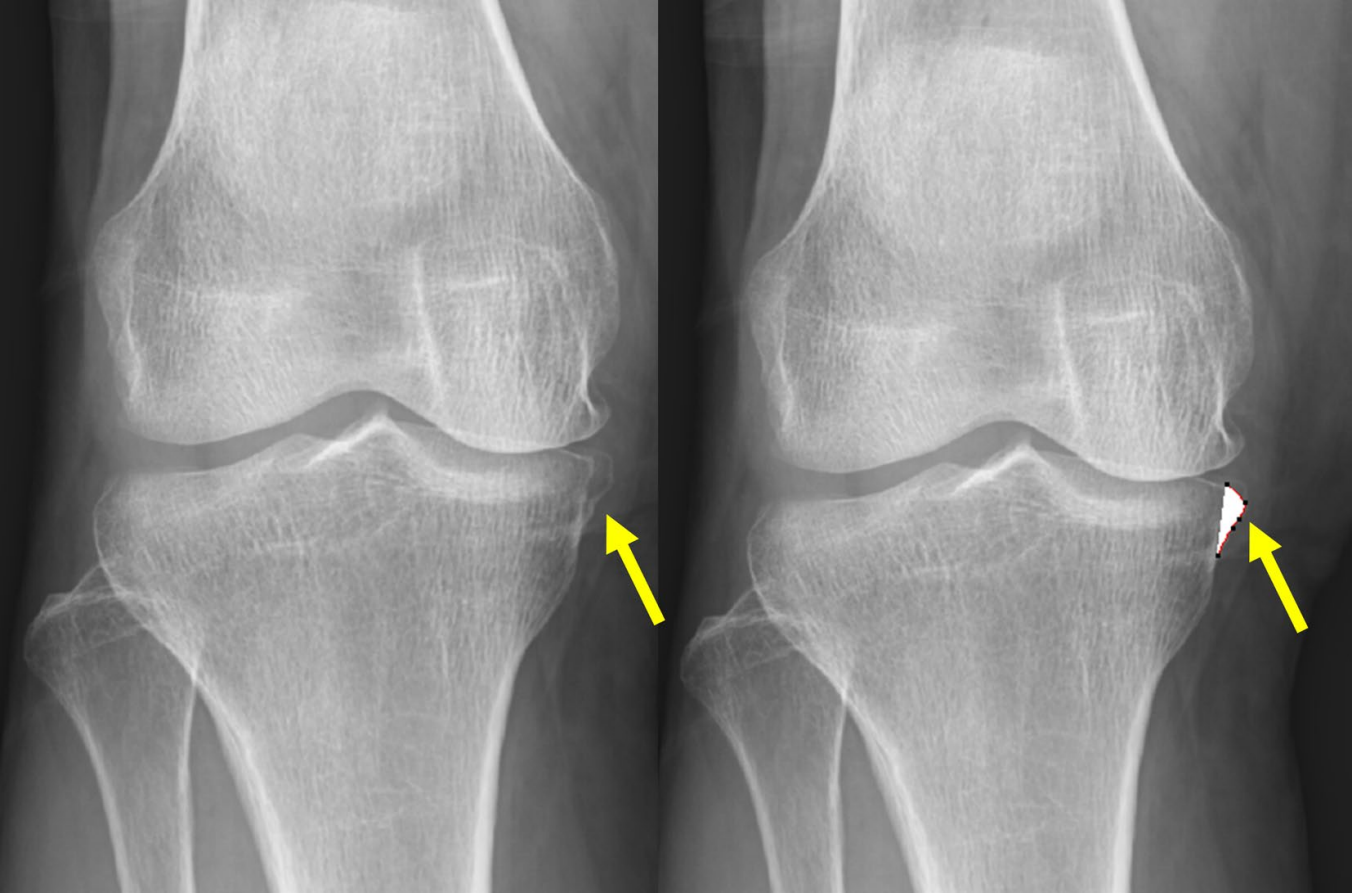
Representative knee frontal radiograph showing the measurement of osteophyte area using the KOACAD system. The system detects the bone margin and calculates the osteophyte area (outlined by the red line) as the medially prominent bony outgrowth beyond the extended contour of the tibia. Arrowhead indicates the osteophyte.

The knee osteophyte area was measured by the Knee Osteoarthritis Computer-Aided Diagnosis (KOACAD) system (INNOTECH Co., Hiroshima, Japan). The KOACAD system was programmed to measure values related to the knee structure, such as joint space narrowing on the medial and lateral sides and osteophyte formation, based on knee morphology obtained from radiographic images. The osteophyte area was calculated as the medially prominent area over the smoothly extended outline of the tibia (Fig. 5)^39^. The KOACAD system demonstrated high reproducibility in an extended position, with an intraclass correlation coefficient of 0.88–0.99^39^. Radiographic assessments were performed by two independent orthopedic surgeons (YS and HK) who were blinded to patient information during the evaluation.

### Statistical analysis

According to previous reports on romosozumab (Cosman, et al.^13^) and denosumab (Cummings et al.^8^), the rate of change in LS-BMD at 12 months in this study was assumed to be 0.1 in the romosozumab group and 0.06 in the denosumab group, with a standard deviation of 0.065 and a significance level of 0.05 (two-sided). To achieve 70% power, 34 cases in one group, with a total of 68 cases in both groups, would be needed. Accordingly, the target sample size was set at 120, accounting for potential dropouts. The required number of cases for analysis was originally calculated to be 86 patients with a power of 80%, and the target sample size was set to be 100 to account for potential dropouts. After the start of the study, two major amendments were made as described in the Study Design: first, the target sample size was increased to 110, and second, the power was decreased to 70%, with the target number of patients set to be 120 as described above.

In this study, no interim efficacy analyses were to be conducted. The stopping criteria were to be applied if the number of enrolled patients did not reach the planned sample size, or if it became apparent that the intervention was clearly effective or ineffective, considering the objectives and content of the clinical trial.

Changes in LS-BMD at 12 months (the primary outcome) and six months (secondary outcome) in the romosozumab group versus the denosumab group were analyzed using analysis of covariance with age, BMI, and baseline BMD as covariates. Estimated means and 95% CIs of TH-BMD, FN-BMD, knee osteophyte area, PROs, grip strength, and BTMs at each time point were analyzed using a linear mixed model with treatment group, time point, and interaction of the treatment group and time point as fixed effects. For comparisons of changes at each time point, the Tukey–Kramer method was used. A multivariate logistic model was used to compare the risk of increased osteophyte area at six months and 12 months between groups. Covariates included baseline osteophyte area, age, BMI, VAS, knee JOA score, OKS, FACIT-fatigue scale, SF-36, EQ-5D, BDI-2, grip strength, BMD, Ca, P, Cre, BTMs, 25(OH)D, and intact PTH. Differences in the proportions of patients with new vertebral fractures, exacerbation of arthritis, and serious adverse events were compared between the romosozumab and denosumab groups using the chi-square test. *P* < 0.05 was considered statistically significant. Stata statistical software, version 18 (Stata Corp LP, College Station, TX, USA), and SAS statistical software, V.9.4 (SAS Institute Corp, Cary, NC, USA), were used for statistical analyses.

### Use of artificial intelligence in manuscript preparation

In the preparation of this manuscript, we utilized ChatGPT, a large language model developed by OpenAI, for assistance with writing and spelling corrections. The model was employed to facilitate the initial drafting and refinement of text, ensuring clarity and grammatical accuracy. However, it is important to note that the responsibility for the content accuracy, data interpretation, and final approval of the manuscript rests solely with the authors. The contributions of ChatGPT were limited to providing language support and did not extend to conceptual development, data analysis, or interpretation of results. Additionally, the authors received English language editing services from ProEdit Japan, Inc.

## Electronic supplementary material

Below is the link to the electronic supplementary material.

Supplementary Material 1

Supplementary Material 2

## Data Availability

The data used and analyzed in this study are available from the corresponding author upon reasonable request.
