# Supplementary figures and images for: A randomized controlled trial comparing romosozumab and denosumab in elderly women with primary osteoporosis and knee osteoarthritis
Source: Sci Rep. 2025 Jul 1;15:22441. doi: 10.1038/s41598-025-05187-7 (PMC12214567; doi:10.1038/s41598-025-05187-7)

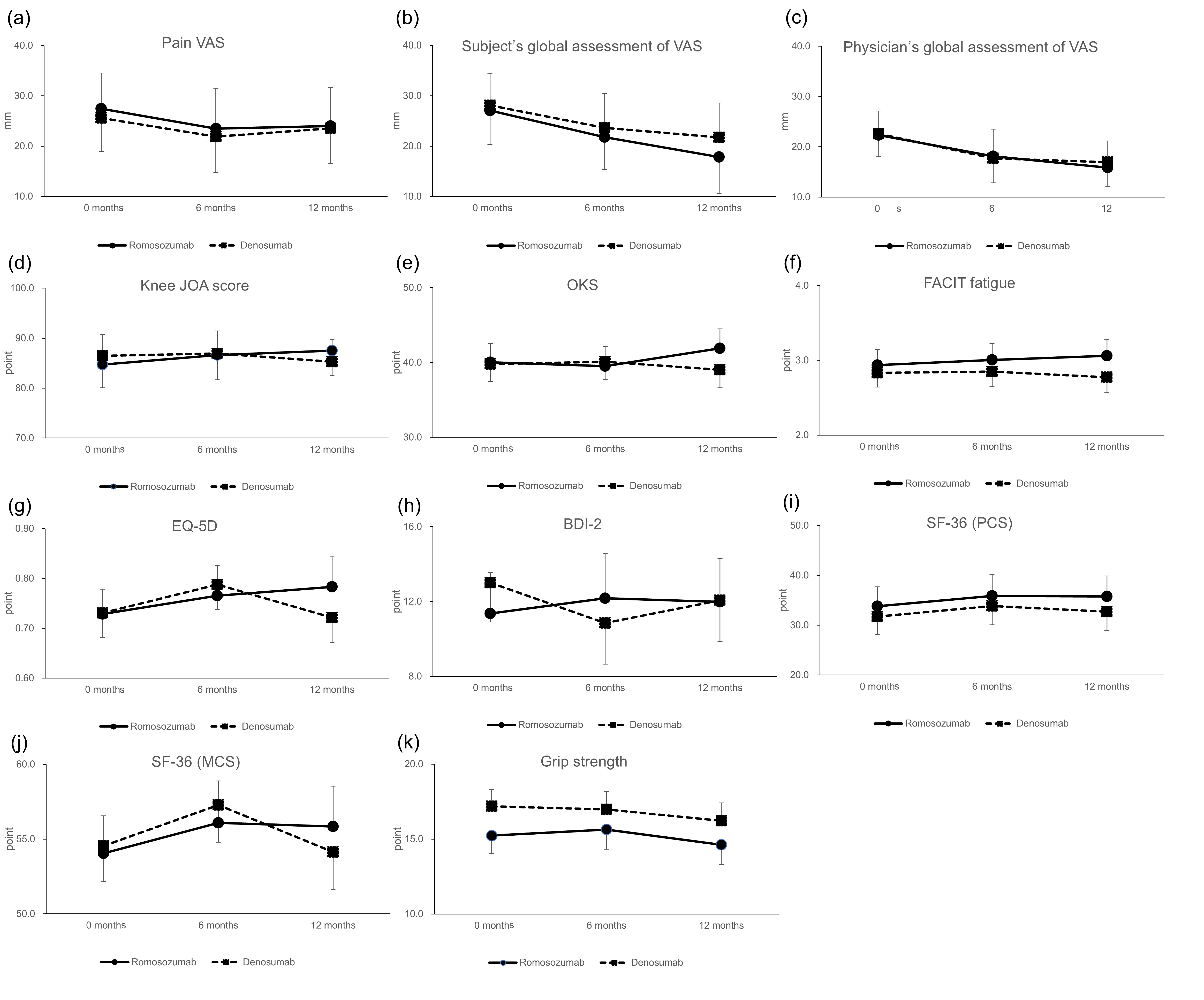

Supplement: Supplementary file 1 — Supplementary Material 1 [file 41598_2025_5187_MOESM1_ESM.tif]
