# Supplementary material for: A randomized controlled trial comparing romosozumab and denosumab in elderly women with primary osteoporosis and knee osteoarthritis
Source: Sci Rep. 2025 Jul 1;15:22441. doi: 10.1038/s41598-025-05187-7 (PMC12214567; doi:10.1038/s41598-025-05187-7)
Supplement: Supplementary file 2 — Supplementary Material 2 [file 41598_2025_5187_MOESM2_ESM.docx]

Supplementary Fig. 1. Estimated mean change in patient-reported outcomes (PROs). (a) Pain visual analog scale (VAS), (b) patient’s global assessment of disease activity VAS, (c) physician’s global assessment of disease activity VAS, (d) patient’s Japanese Orthopedic Association (JOA) score, (e) Oxford Knee Score (OKS), (f) Functional Assessment of Chronic Illness Therapy (FACIT)-fatigue scale, (g) EQ-5D-5L (EQ-5D), (h) the second edition of the Beck Depression Inventory (BDI-2), (i) SF-36 (Physical Component Summary), (j) SF-36 (Mental Component Summary), and (k) grip strength. Error bars represent 95% confidence intervals. No significant difference was observed between the romosozumab and denosumab groups.
